# Varicose Veins and Their Complications.—II

**Published:** 1897-10-16

**Authors:** W. McAdam Eccles

**Affiliations:** Assistant Surgeon, West London Hospital, City of London Truss Society, &c.


### VARICOSE VEINS AND THEIR COMPLICATIONS.—II.

By W. McAdam Eccles, M.S.(Lond.), F.R.C.S.(Eng.), Assistant Surgeon, West London Hospital, City of London Truss Society, &c.

There is no doubt that a considerable majority of those who are the subjects of varicose veins have but few, if any, complications as a result of their venous trouble, and those complications which may happen to exist are but slight. Indeed, some authorities go so far as to say that the larger number of the conditions which are usually described as sequelæ of varicosity have but little real connection with it. It may be readily granted that many persons without varicose veins of the lower extremity may suffer from a very troublesome local eczema, and it is equally true that many others, who have dilated veins, have no eczema. However, taking all these facts into consideration, I cannot myself but feel that varicosity of the veins of the leg is at least a very potent predisposing cause of some very serious conditions.

There can be no gainsay to the fact that the state of the vein when it is varicosed renders it more liable to attacks of inflammation than a healthy vein would be. Such inflammations may be of a simple nature, generally the outcome of mechanical injury, without lesions of the skin, or they may be of a septic nature, usually following the entrance of micrococci through a wound or from an ulcer.

The simple form of inflammation is particularly likely to occur where the dilated vessels are left unsupported and have been subjected to local compression or bruising. Thus phlebitis is common in these cases in the lower part of the leg, which is necessarily more exposed to injury than the higher parts; and, again, it has been my observation that the pressure and, perhaps, irritation of a band such as a garter, or piece of tape, so often used by women, will determine the commencement and the site of the inflammation. The region surrounding the inflamed vein quickly becomes very painful and tender, and the vein itself soon has the blood within it coagulated; in other words, a thrombus appears. This in the earlier stages will be soft and easily detachable, but before long it becomes much harder and firmly attached to the vein wall. No doubt when first formed there is some likelihood of the clot being carried towards the heart, and producing embolism, so possibly causing blockage of the pulmonary artery and consequent death. The treatment of the simple inflammation of a dilated vein resolves itself into absolute rest of the part for at least ten days, and relief of the pain by the application of the extract of belladonna with glycerine over the line of the vein, covering this with a flannel wrung out of very hot water.

In the septic form of inflammation a much more serious condition is present. Here the entrance of cocci produces not only inflammation of the vein itself, but in addition a considerable amount of periphlebitis, and it must be remembered that there is always some danger of general infection—pyæmia in other words—through the circulation.

By no means infrequently suppuration in the tissues around the vein occurs, and a localised abscess is formed.

In dealing with a case where the veins have become infected, it is well as the first step in the treatment to prevent further infection—to promptly render aseptic the area in which the micro-organisms have been inoculated. Afterwards the region of the vein should be covered with belladonna and glycerine beneath a fomentation. It might perhaps in some cases be advisable to ligature the vein at a healthy spot at some distance on the proximal side of the inflamed vein, in order to obviate any possibility of the staphylococci being carried into the general circulation. If an abscess forms or cellulitis supervenes, one or more free incisions will be necessary, together with effective cleansing of the suppurating area. Boracic fomentations are the best after-treatment. Unless such measures are promptly and energetically carried out very serious consequences may ensue.

It is very questionable whether a vein once thrombosed ever becomes pervious again. In some cases the blockage of the vein or veins seems to act beneficially on the varicose conditions, but in others it merely aggravates the already troublesome lesion from which the patient is suffering. Rarely a phlebolith is found in old varicose veins, due to calcification of the vein wall or of the enclosed clot.

I now pass to what I am accustomed to speak of as the sequence of events in the formation of the so-called varicose or callous ulcer of the leg.

The dilated condition of the veins, of which the chief function is to return the blood from the part, leads in the greater number of cases to more or less congestion and œdema of the tissues. The nutrition of the skin is thereby seriously impaired, and a chronic form of eczema is liable to supervene—the varicose eczema so frequently seen in our hospital out-patients' rooms. This eczema but seldom has any tendency to abundant weeping, owing to the paucity of vesicles, but what amount of discharge there is accumulates in scales or scabs on the surface, together with a considerable amount of extraneous matter.

Severe irritation is usually present, partly as the outcome of the actual dermatitis and partly owing to the heaped up dirt on the skin. The patient cannot refrain from scratching the part in order to obtain some temporary relief, and in performing this act is very liable to produce some slight abrasions. In many cases such may quickly become covered with epithelium; but the open wound is ready to receive infection, and in far too many instances it does so. The filthy accumulation on the skin of discharge, dried ointment, dirt and micro-organisms, especially staphylococci, is almost certain to be inoculated. A septic ulcer has thus its starting point, and unless dealt with at the very commencement is sure to extend.

Such an ulcer is very often called a varicose ulcer, thereby definitely connecting it with a varicose condition of the veins of the part. It is very true that this term is not scientifically a sound one, but I am bound to confess that it is one which conveys a considerable amount of meaning. Probably if there had been no dilated veins in the region, no congestion would have been present, and no consequent failure in nutrition, and therefore no resulting eczema. Thus the absence of the irritation and the scratching, and so no likelihood of a wound. And again, the want of proper return of venous blood seems to have a very definite effect upon the character of the ulcer itself, and upon the rate of its disappearance under treatment. On the other hand, one must realise that the chief factor in the production of these ulcers is not varicosity, but septicity. I venture to affirm this very strongly, and to urge that, in order to obtain a satisfactory healing, asepsis must be secured above all things.

The usual plan of prescribing ointments and nothing more in these cases is not only unscientific, but practically worthless, and even harmful. In a former paper,* I have fully dealt with the rational treatment of septic ulcers associated with varicose veins of the leg, and I shall, therefore, only briefly allude to it here.

In the first place, the ulcers and their surroundings must be thoroughly cleansed with soap and water liberally used. This may cause a considerable amount of pain, but is most essential. Next the surface of the ulcer is to be irrigated with an antiseptic, of which 1-1,000 perchloride of mercury answers well. A covering of antiseptic gauze is forthwith applied by rolling

* The Hospital, September, 1896, p. 424.

on a carbolic gauze bandage from the foot to the knee, with several layers over the actual seat of the ulcer. Outside this is painted on while hot a modified Unna's paint, which consists of zinc oxide, boracic acid, gelatine, glycerine, and water. Gauze is again applied over this, and finally a webbing bandage from the foot to the knee outside all.

The dressing for the first time should not be left on longer than three days, but in a short while, as the wound becomes aseptic, it may remain undisturbed for a fortnight or more. The surface of the ulcer soon becomes covered with healthy granulations, and healing is usually rapid.

There is no need for patients to be confined to bed during this treatment.

Such ulcers have lately been treated by oxygen; but, while waiting for a more extended trial to bring a more definite knowledge of results, I confess that I think there are several great objections to this method of treatment. It requires special apparatus of a cumbersome nature; requires usually that a patient be in bed and therefore in enforced idleness; and, lastly, it is decidedly expensive. I believe myself it chiefly acts by bringing about asepsis, which can be equally well obtained by less involved methods.

